# Transbronchial biopsy results according to diffuse interstitial lung disease classification. Cryobiopsy *versus* forceps: MULTICRIO study

**DOI:** 10.1371/journal.pone.0239114

**Published:** 2020-09-21

**Authors:** Virginia Pajares, Manuel Núñez-Delgado, Gloria Bonet, Javier Pérez-Pallarés, Raquel Martínez, Noelia Cubero, Txomin Zabala, Rosa Cordovilla, Javier Flandes, Carlos Disdier, Alfons Torrego, in collaboration with MULTICRIO Group researchers

**Affiliations:** 1 Respiratory Medicine, Hospital de la Santa Creu i Sant Pau, Barcelona, Spain; 2 Biomedical Research Institute Sant Pau (IIB-Sant Pau), Barcelona, Spain; 3 Respiratory Medicine, Hospital Universitario Álvaro Cunqueiro, Vigo, Spain; 4 Respiratory Medicine, Hospital Universitario de Germans Trias i Pujol, Barcelona, Spain; 5 Respiratory Medicine, Hospital Universitario de Santa Lucía, Cartagena, Murcia, Spain; 6 Respiratory Medicine, Hospital Universitario La Fé, Valencia, Spain; 7 Respiratory Medicine, Hospital Universitario de Bellvitge, Barcelona, Spain; 8 Respiratory Medicine, Hospital Galdakao-Usansolo, Vizcaya, Bizkaia, Spain; 9 Respiratory Medicine, Hospital Universitario de Salamanca, Salamanca, Spain; 10 Respiratory Medicine, Fundación Jiménez Díaz, Madrid, Spain; 11 Respiratory Medicine, Hospital Universitario de Valladolid, Valladolid, Spain; University of Texas McGowan Medical School at Houston, UNITED STATES

## Abstract

**Background:**

In recent years, transbronchial cryobiopsy (TBCB) has come to be increasingly used in interventional pulmonology units as it obtains larger and better-quality samples than conventional transbronchial lung biopsy (TBLB) with forceps. No multicenter studies have been performed, however, that analyse and compare TBCB and TBLB safety and yield according to the interstitial lung disease (ILD) classification.

**Objectives:**

We compared the diagnostic yield and safety of TBCB with cryoprobe sampling versus conventional TBLB forceps sampling in the same patient.

**Method:**

Prospective multicenter clinical study of patients with ILD indicated for lung biopsy. Airway management with orotracheal tube, laryngeal mask and rigid bronchoscope was according to the protocol of each centre. All procedures were performed using fluoroscopy and an occlusion balloon. TBLB was followed by TBCB. Complications were recorded after both TBLB and TBCB.

**Results:**

Included were 124 patients from 10 hospitals. Airway management was orotracheal intubation in 74% of cases. Diagnostic yield according to multidisciplinary committee results for TBCB was 47.6% and for TBLB was 19.4% (p<0.0001). Diagnostic yield was higher for TBCB compared to TBLB for two groups: idiopathic interstitial pneumonias (IIPs) and ILD of known cause or association (OR 2.5; 95% CI: 1.4–4.2 and OR 5.8; 95% CI: 2.3–14.3, respectively). Grade 3 (moderate) bleeding after TBCB occurred in 6.5% of patients compared to 0.8% after conventional TBLB.

**Conclusions:**

Diagnostic yield for TBCB was higher than for TBLB, especially for two disease groups: IIPs and ILD of known cause or association. The increased risk of bleeding associated with TBCB confirms the need for safe airway management and prophylactic occlusion-balloon use.

**Trial registration:**

**clinicaltrials.gov identifier:** NCT02464592.

## Introduction

Transbronchial cryobiopsy (TBCB), which uses modified cryotherapy probes, resulted from the need to improve the diagnostic yield of conventional transbronchial lung biopsy (TBLB). Flexible cryotherapy probes as currently used, with greater freezing power and speed than rigid probes, allow for increased traction on the tissue, which results in larger specimens. Studies that have analysed histological material obtained by cryoprobe for endobronchial tumours have observed that, since samples are both larger than those obtained using a conventional biopsy and histologically better preserved, they facilitate histological diagnosis and the application of immunohistochemical techniques [1, 2]. Those observations have led to increased use of the cryoprobe as an alternative to the conventional biopsy technique for the purpose of studying diffuse lung diseases [3–6].

However, there are important methodological differences in the mainly retrospective single-centre studies of TBCB to date, in terms of both technical and experimental design. Diagnostic yields, while superior to those of conventional TBLB, vary between 50.6% and 100% depending on the study [7]. To date, only one randomized clinical trial (conducted in a single centre) has compared TBCB and TBLB and analysed the complications associated with each [6], while no prospective multicenter study has assessed diagnostic yield according to diffuse interstitial lung disease (ILD) classes or has assessed the risk-benefit profile of both techniques performed in turn in the same patient.

This study was designed to determine, for the same patient with clinical and radiographic findings of ILD, the diagnostic yield and safety of TBCB in comparison with TBLB using a conventional forceps. The procedures using different airway managements were also compared.

## Patients and methods

### Study design

Prospective multicenter study undertaken in 10 interventional pulmonology units between April 2014 and June 2017. Patients with ILD but no definitive diagnosis referred for lung biopsy were included. This indication was performed independently of the purpose of the study by an expert ILD physician based on clinical and radiological findings (HRCT, high-resolution computed tomography). Based on current guidelines, a lung biopsy was requested to establish a definitive diagnosis. All included patients signed an informed consent prior to their inclusion in the study.

### Population

Inclusion criteria were patients aged 18–80 years, a HRCT pattern indeterminate or alternative for UIP and absence of definitive diagnosis. Exclusion criteria were definite or probable UIP pattern in the HRCT scan, use of anticoagulation therapy, presence of a coagulation disorder (thrombocytopenia <50000 cells/mm3, abnormal platelet count >1 million cells/mm3, international normalized ratio (INR) >1.5, and activated partial thromboplastin time >50 s), and unstable heart disease (uncontrolled cardiac arrhythmia or active myocardial ischaemia). Other exclusion criteria were hypoxaemia (partial pressure of oxygen in arterial blood < 60 mmHg), severe respiratory impairment (forced expiratory volume in 1 s (FEV_1_) < 50%, total lung capacity (TLC) < 50%, diffusing capacity of carbon monoxide (DLCO) < 40% of reference), and unstable heart disease (uncontrolled cardiac arrhythmia, active myocardial ischaemia).

Data available for the patients included complete blood count, serum biochemistry and coagulation tests, lung function including forced expiratory volume in 1 s (FEV_1_), total lung capacity (TLC) and diffusing capacity of carbon monoxide (DLCO), and thoracic high-resolution computed tomography (HRCT). Echocardiography was performed as necessary.

### Protocol

Patients scheduled for the intervention–TBLB followed by TBCB in that order–in the pulmonology units of the participating hospitals were sedated and monitored by the anaesthesiologist attached to each unit. Airway management–with laryngeal mask, orotracheal tube or rigid bronchoscope–was that reflected in the protocol of each centre. All biopsies were fluoroscopy-guided to zones preselected in accordance with radiological involvement. An occlusion balloon was placed before performing biopsies and the conventional forceps or cryoprobe was positioned 1–2 cm from the visceral pleura.

The goal was to obtain a minimum of three conventional TBLBs, followed–provided there were no complications that contraindicated additional biopsies–by a minimum of three TBCBs. Bleeding was recorded according to the following classification: grade 0 (no bleeding, i.e., no observed blood remains; grade 1 (mild bleeding, i.e., some observed blood remains but endoscopic intervention not necessary); grade 2 (slight bleeding, i.e., bleeding requires balloon occlusion and stops in <3 minutes; grade 3 (moderate bleeding, i.e., bleeding requires balloon occlusion, needs >3 minutes to bring under control and requires suspension of the procedure; and grade 4 (severe bleeding, i.e., bleeding is endoscopically uncontrollable, causes haemodynamic or respiratory instability and requires suspension of the procedure and/or other invasive haemostatic interventions). Pneumothorax occurrence was assessed by means of fluoroscopy after the biopsies obtained using each method. Biopsies were performed in several bronchial segments and lobes of the lung that had previously been identified as affected by thoracic HRCT. All bleeding and pneumothorax complications were recorded.

Recorded for all patients, prior to commencing the intervention, were blood pressure, heart and respiratory rates, oxyhaemoglobin saturation and electrocardiogram and capnography data. All samples were harvested using a flexible videobronchoscope in procedures that included endoscopic exploration and other diagnostic tests as required.

Conventional TBLB was performed using a 2-mm biopsy forceps (Biopsy Forceps Boston ® Ref: 1556, Olympus^®^ Ref: FB-19E, Biopsy Forceps Medi-Inn Ref BP-40144). The TBCB was performed using a 900-mm long flexible cryoprobe with diameter 2.4 mm (Ref: 20426–032) connected to a cryotherapy unit (Erbokryo® CA). Freezing was applied for 3–4 seconds after which the bronchoscope was withdrawn. Once intervention was complete, the patient remained under observation and was discharged if no complications were evident.

### Sample processing

Extracted biopsy specimens were fixed in 4% paraformaldehyde and embedded in paraffin; 4-μm sections were processed for the staining protocol (hematoxylin and eosin, Masson’s trichrome to identify collagen and Orcein stain to detect elastic fibre). Histological analyses were performed by the pathologists attached to each centre.

### Histological assessment

After transbronchial biopsy, the pathologist received clinical and radiological information about the case. Both, TBLB and TBCB, were evaluated simultaneously. The pathologists classified the samples according to certainty of diagnosis, as follows: diagnostic pattern, if the sample contained histologic findings characteristic of a particular form of ILD or if there were nonspecific findings consistent with clinical and radiologic findings suggestive of a disease; or nondiagnostic pattern, when the findings did not suggest any particular disease or when the material harvested was less than 1 mm in diameter.

Tissue quality variables were recorded according to an objective protocol and agreement regarding each individual pathologist’s identification of the histological pattern [8].

### Multidisciplinary diagnosis

Finally, all cases were discussed at the Multidisciplinary Meeting (MDM) of each center. First, clinical and radiological findings were presented. Subsequently the histology was added. The MDM evaluated each technique sample and how it contributed to the diagnosis. We categorized a biopsy as diagnostic only in those cases were the TBCB or TBLB findings were able to contributed to a definitive or high confidence diagnosis.

After MDM, the final diagnostics established with conventional TBLB and TBCB were classified according to Spanish guidelines [9] and the ATS/ERS/JRS/ALAT International Multidisciplinary Consensus Classification [10, 11]. Four disease groups were established–labelled groups 1, 2, 3 and 4, respectively–as follows: idiopathic interstitial pneumonias (IIPs); ILD of known cause or association; granulomatous ILD and miscellaneous ILD; and other diagnoses (non-ILD).

### Statistical analysis

Data were analysed using the SPSS statistical package, version 25 for Windows (SPSS Inc, Chicago IL, USA). Results are reported as means and standard deviations for the quantitative variables and as absolute values and percentages for the qualitative variables. The student-t test was used to compare the two biopsy techniques. For the categorical variables, a logistic regression was performed to estimate odds ratio (OR) values along with their 95% confidence interval (CI) and a statistical significance level of 0.05.

### Ethics statement

The study was conducted in compliance with the Declaration of Helsinki and was evaluated and approved by the clinical research ethics committee (CEIC) of each hospital (CEIC reference: IIBSP-CRI-2014-05). The study was registered at ClinicalTrials.gov (NCT02464592).

## Results

A total of 124 patients were included (Table 1). A pre-intervention echocardiogram was not available for 38% of the patients; in 24% of the remaining patients, ultrasound abnormalities were observed. In 2.9% of the cases, mild to moderate pulmonary arterial hypertension (mean 25-55mmHg) was recorded.

**Table 1:** Baseline characteristics of 124 included patients with suspected ILD.

Variable	
Sex (male/female)	72/52
Age (years)	65.7±11.9
BMI (kg/m^2^)	28.8±4.2
FVC (L)	2.2 ±12
FVC (% ref)	79±20
FEV_1_ (L)	1.7±10
FEV_1_ (% ref)	80±18
FEV_1_/FVC (% ref)	80.2±8.7
TLC (% ref)	76.6±18.5
DLCO, % ref	57.7±17.4
INR	0.9±0.3
Platelets (x10^9^/L)	214.1±74

Data are presented as mean ± standard deviation, unless otherwise indicated.

% ref, percentage of the reference value. BMI, body mass index; FEV_1_, forced expiratory volume in 1 second; FVC, forced vital capacity; TLC, total lung capacity; DLCO, carbon monoxide diffusing capacity; INR, international normalized ratio.

With respect to airway management, orotracheal intubation was performed in 74% of the patients. The time required for biopsies was 15.2±8.1 minutes for TBCB compared to 12.1±8.4 minutes for conventional TBLB (p<0.001). Biopsy zones were selected according to radiological involvement. Only 8% of TBCB and TBLB biopsies were performed in the upper lobes.

### Diagnostic yield

A total of 942 lung biopsy specimens were evaluated, 508 TBLB and 434 TBCB. The mean number of biopsies per patient for TBCB was lower than for TBLB (3.5 vs 4.1; p<0.001).

**Fig 1:**
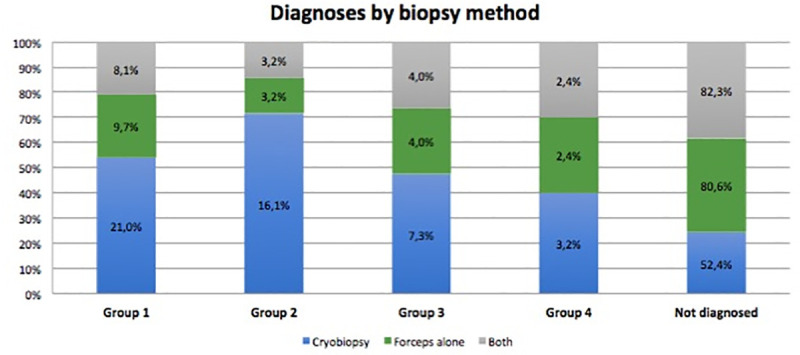
Percentage of diagnoses for each method and percentage of patients not diagnosed by either of the two biopsy methods.

To evaluate diagnostic yield, diagnostic and non-diagnostic patterns were classified separately. The diagnostic yield according to histopathology results was higher for TBCB than for TBLB (54.8% vs. 19.4%; p<0.0001), with TBCB proving to be five times more diagnostically effective than TBLB (OR 5.1; 95% CI: 3.2–7.9; p<0.0001). Likewise, according to the MDT, the diagnostic yield was four times higher for TBCB than for TBLB (47.6% vs. 19.4%; OR 3.8; 95% CI: 2.5–5.8; p<0.0001) (Table 2). Fig 1 show diagnoses with each biopsy method and non diagnoses. As for multidisciplinary committee diagnoses by disease groups, for groups 1 and 2, the diagnostic yield for TBCB was higher than for TBLB: in group 1, 21% of patients were diagnosed by TBCB vs 9.7% by TBLB, indicating that TBCB was 2.5 times more diagnostically effective than TBLB (OR 2.5; 95% CI: 1.4–4.2; p = 0.0008), and in group 2 the differences were also greater in favour of TBCB, which was 5 times more diagnostically effective than TBLB (OR 5.8; 95% CI: 2.3–14.3; p = 0.0002). No significant differences were observed in diagnostic yield for groups 3 and 4. Tables 3 and 4 show the distributions of pathologies in groups. The multivariate analysis maintained the diagnostic yield differences for the different airway managements.

**Table 2:** Multidisciplinary Meeting (MDM) diagnoses by biopsy technique.

MDM diagnostic	Conventional forceps N = 124	Cryoprobe N = 124
Idiopathic pulmonary fibrosis	3 (2.4%)	10 (8.1%)
RB ILD-associated	4 (3.2%)	6 (4.8%)
Non-specific interstitial pneumonia	3 (2.4%)	5 (4.0%)
Organizing pneumonia	1 (0.8%)	4 (3.2%)
Desquamative interstitial pneumonia	1 (0.8%)	1 (0.8%)
Hypersensitivity pneumonitis	3 (2.4%)	13 (10.5%)
Drug-induced pneumonitis	1 (0.8%)	3 (2.4%)
Lipoid pneumonia	-	2 (1.6%)
Connective tissue disease-associated ILD	-	2 (1.6%)
Sarcoidosis	4 (3.2%)	6 (4.8%)
Pulmonary amyloidosis	1 (0.8%)	1 (0.8%)
Langerhans cell histiocytosis	-	1 (0.8%)
Lymphangioleiomyomatosis	-	1 (0.8%)
Lung neoplasm	3 (2.4%)	3 (2.4%)
Lymphoproliferative syndrome	-	1 (0.8%)
**Total diagnoses**	100 (80.6%)	65 (52.4%)
**Total non-diagnoses**	24 (19.4%)	59 (47.6%)
OR (Cryo/Conv)	3.7821	
[95% CI]	[2.4546–5.8274]	
P-value	<0.0001	

OR, odds ratio; CI, confidence interval (upper and lower limits in square brackets); RB, respiratory bronchiolitis; ILD, interstitial lung disease.

**Table 3:** Diagnostic yield for multidisciplinary committee review of diagnoses by biopsy technique.

	Conventional forceps	Cryoprobe
	N = 124	N = 124
**GROUP 1. IIPs**	12 (9.7%)	26 (21.0%)
OR (Cryo/Conv)	2.4762	
[95% CI]	[1.4543–4.2160]	
P-value	0.0008	
**GROUP 2. ILD of known cause or association**	4 (3.2%)	20 (16.1%)
OR (Cryo/Conv)	5.7692	
[95% CI]	[2.3269–14.3038]	
P-value	0.0002	
**GROUP 3. Granulomatous ILD and miscellaneous ILD**	5 (4.0%)	9 (7.3%)
OR (Cryo/Conv)	1.8626	
[95% CI]	[1.0127–3.4257]	
P-value	0.0454	
**GROUP 4. Other non-ILD diagnoses**	3 (2.4%)	4 (3.2%)
OR (Cryo/Conv)	1.3444	
[95% CI]	[0.7528–2.4009]	
P-value	0.3171	

OR, odds ratio; CI, confidence interval (upper and lower limits in square brackets); IIP, idiopathic interstitial pneumonia; ILD, interstitial lung disease.

**Table 4:** Multidisciplinary committee diagnoses by biopsy technique according to diffuse interstitial classification.

**GROUP 1. Idiopathic interstitial pneumonias**		
	Conventional	Cryoprobe
	N = 12	N = 26
Idiopathic pulmonary fibrosis	3 (2.4%)	10 (8.1%)
RB ILD-associated	4 (3.2%)	6 (4.8%)
Non-specific interstitial pneumonia	3 (2.4%)	5 (4.0%)
Organizing pneumonia	1 (0.8%)	4 (3.2%)
Desquamative interstitial pneumonia	1 (0.8%)	1 (0.8%)
**GROUP 2. ILD of known cause or association**		
	Conventional	Cryoprobe
	N = 4	N = 20
Hypersensitivity pneumonitis	3 (2.4%)	13 (10.5%)
Drug-induced pneumonitis	1 (0.8%)	3 (2.4%)
Lipoid pneumonia	0	2 (1.6%)
Connective tissue disease-associated ILD	0	2 (1.6%)
**GROUP 3. Granulomatous ILD and miscellaneous ILD**		
	Conventional	Cryoprobe
	N = 5	N = 9
Sarcoidosis	4 (3.2%)	6 (4.8%)
Pulmonary amyloidosis	1 (0.8%)	1 (0.8%)
Langerhans cell histiocytosis	0	1 (0.8%)
Lymphangioleiomyomatosis	0	1 (0.8%)
**GROUP 4. Other non-ILD diagnoses**		
	Conventional	Cryoprobe
	N = 3	N = 4
Lung neoplasm	3 (2.4%)	3 (2.4%)
Lymphoproliferative syndrome	0	1 (0.8%)

Data are presented as numbers and percentages. RB, respiratory bronchiolitis, IIP, idiopathic interstitial pneumonia; ILD, interstitial lung disease.

### Complications

No bleeding was observed after conventional TBLB in 56.4% of patients compared to 44% of patients after TBCB (p<0.0001); overall, fewer bleeding episodes (all grades) were observed for conventional TBLB compared to TBCB. The most frequent bleeding associated with either technique was grade 1 bleeding, at 29.8% for conventional TBLB vs 42.1% for TBCB. Grade 3 bleeding after TBCB was 6.5% compared to 0.8% after conventional TBLB, while there was just a single case of grade 4 bleeding (0.8%), resulting from TBCB and requiring intubation and intensive care; this patient died 40 days after the procedure for reasons attributed to diffuse ILD progression. The multivariate logistic regression analysis indicated that differences in airway management did not modify the bleeding risk (any grade) after TBCB.

Three cases of pneumothorax (2.4% of patients) were associated with TBCB, in two cases requiring a drainage tube. A single pneumomediastinum that was detected late was associated with both techniques (Table 5). Upper lobe biopsies did not result in an increase in pneumothorax cases for either of the techniques.

**Table 5:** Number of complications according to biopsy technique.

	Conventional	Cryoprobe	P
	N = 124	N = 124	
No bleeding	70 (56.4%)	44 (35.5%)	<0.0001
Bleeding			
Grade 1	36 (29.8%)	51 (42.1%)	
Grade 2	17 (14.0%)	21 (17.4%)	
Grade 3	1 (0.8%)	8 (6.5%)	
Grade 4	0	1 (0.8%)	
Pneumothorax	0	3 (2.4%)	
Pneumomediastinum	1 (0.8%)	1 (0.8%)	

Data are presented as numbers and percentages.

## Discussion

Our findings demonstrate that, in patients with suspected diffuse ILD, diagnostic yield is significantly higher for TBCB than for conventional TBLB, after both histological evaluation and multidisciplinary committee evaluation. Regarding the diagnoses, a novel finding was in relation to the distribution of pathologies according to diffuse ILD classes [11], as TBCB resulted in higher diagnostic yields for the IIPs and ILD of known cause or association (groups 1 and 2). More specifically, TBCB diagnosed more cases of UIP, non-specific interstitial pneumonia (NSIP) and hypersensitivity pneumonitis (HP).

Within the group of IIPs, it was necessary to distinguish the UIP pattern, related mainly to idiopathic pulmonary fibrosis (IPF), given the important prognostic implications and currently available treatment options [12].

Several authors have questioned the usefulness of conventional TBLB to diagnose certain interstitial diseases, including UIP [13–15]. The results of our study, which are consistent with those reported for other series [16–19], would indicate TBCB to be a useful technique for the diagnosis of IIPs, as proposed in the Fleischner Society white paper [20]. Recently, Troy et al. [21] showed in a prospective, multicenter study (COLDICE) high levels of agreement between TBLC and surgical lung biopsy for histopathological and multidisciplinary diagnoses. Despite this, TBCB is still not included in the diagnostic algorithm of the latest ATS/ERS/JRS/ALAT Guideline for IPF diagnosis [22], while no consensus has been reached regarding a favourable recommendation for TBCB in IPF diagnosis, especially in centres without experience [22, 23].

In relation to groups 3 (granulomatous ILD and miscellaneous ILD) and 4 (other non-ILD diagnoses), while diagnostic yield for the two techniques were similar, the multidisciplinary committee found that TBCB more frequently diagnosed certain histologically complex entities than did conventional TBLB, specifically, Langerhans cell histiocytosis, lymphangioleiomyomatosis and parenchymal involvement associated with a lymphoproliferative syndrome. As for entities such as sarcoidosis and diseases whose distribution or radiological pattern is such that histological diagnosis is difficult using conventional TBLB, our findings–while they do not allow for a categorical recommendation in this regard, given the limited number of cases–would advise, before indicating TBCB, preliminary comparative risk-benefit evaluations for the two techniques, as well as thorough radiological evaluation.

Our findings reflect a lower percentage of histological and multidisciplinary diagnoses obtained by TBCB compared to other studies. One explanation for this lower diagnostic yield is the wide range of experience with the technique–by both the participating hospitals and the multidisciplinary committee members. Nonetheless, the prospective and multicenter design of the study would suggest that the results obtained are transversal and reflect the current healthcare reality. In relation to the determination of committee composition, Castillo et al [24] recently reviewed criteria regarding standardization of the necessary professional experience. As for experience, it has been demonstrated that diagnostic yield reflect the TBCB learning curve [25]. Our results reflect TBCB diagnostic yield variability, documented as between 50.6% and 100% in the recently published expert consensus [7]. Our results are also consistent with those reported for a single-centre randomized clinical trial [6] and for other published studies [26–28].

**Fig 2:**
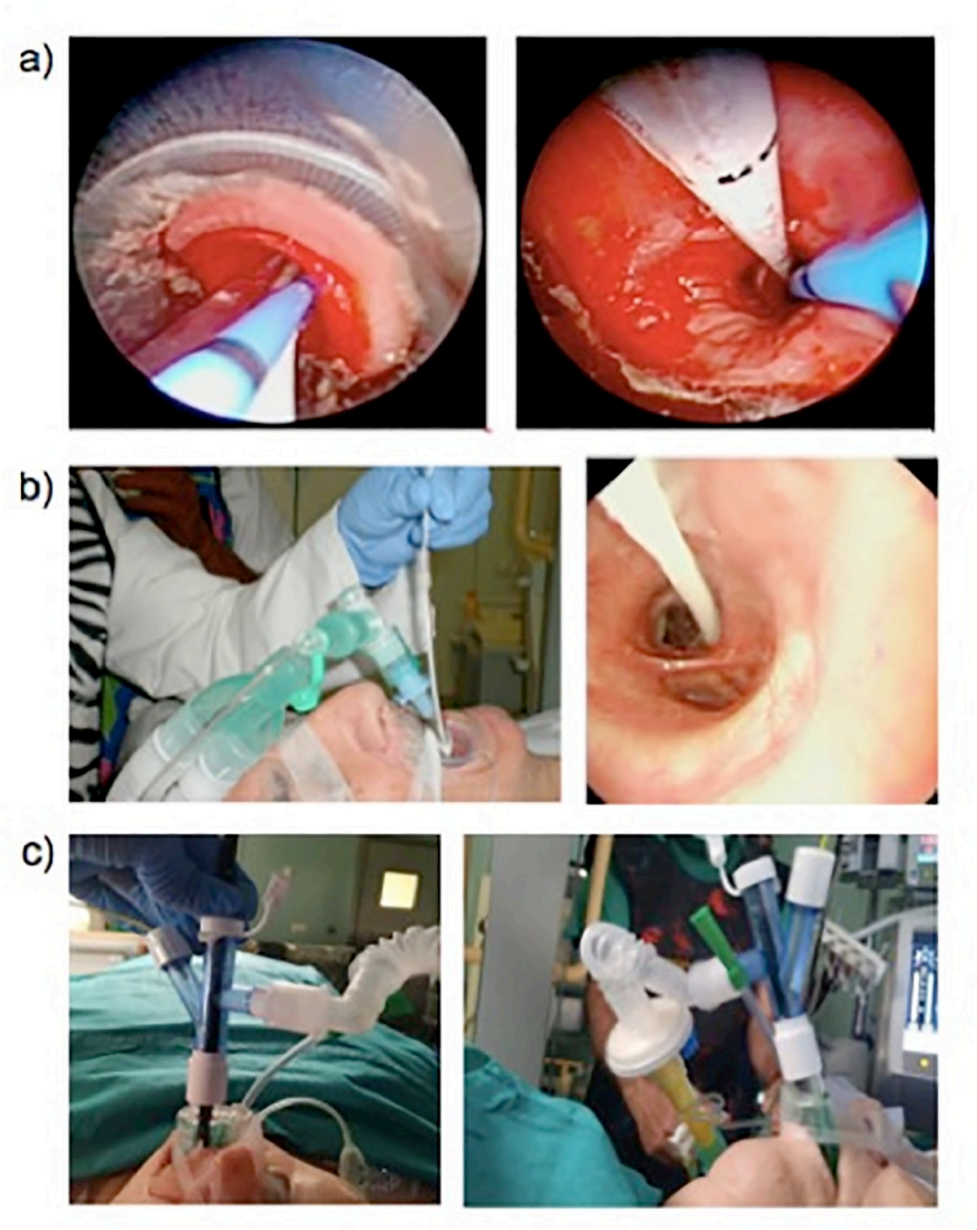
Different airway management approaches to transbronchial biopsy with cryoprobe and placement of the occlusion balloon. (a) Intubation using a bronchoscope and a flexible endotracheal tube (Bronchoflex 7.5 mm, Rüsch, Teleflex Medical, Durham, NC, USA). (b) Intubation and occlusion balloon insertion using a rigid bronchoscope. (c) Intubation and occlusion balloon insertion using a laryngeal mask.

With respect to airway management (Fig 2), orotracheal intubation was the main technique used in our study, although use of a laryngeal mask or rigid bronchoscope did not affect biopsy duration, the number of biopsies or the number or severity of complications observed. While the recommendation of a group of experts is to use orotracheal intubation [7], other authors have used the laryngeal mask or rigid bronchoscope techniques [29, 30] with no major difference in complications or limitations.

In relation to safety, complications were bleeding and pneumothorax. More grade 3 bleeding was observed after TBCB, brought under control in all cases by a previously inserted occlusion balloon. One case of grade 4 bleeding associated with TBCB required selective bronchial intubation, while no deaths associated with bleeding were recorded. These results are similar to those reported by other authors. A meta-analysis by Johannson et al. [31] point to great heterogeneity in describing and classifying bleeding, as the rate of moderate to severe bleeding was 39%, while the range was 0% to 78%. Sharp et al. [32], reported that three deaths occurred due to bleeding after TBCB (0.5%). It should be noted, however, that there is an absence of consensus regarding bleeding definition and quantification and, hence, the highly variable findings of different studies cannot easily be compared. Since we classified bleeding according to the endoscopic interventions necessary to bring it control, we suggest that our assessment of the endoscopic and clinical relevance of bleeding was objective.

Our study has certain limitations. First, the pathologists were not blinded as to sampling technique. However, they were required to record tissue quality variables according to an objective, pre-established protocol and agreement regarding individual pathologist’s identification of the histological pattern. All cases were discussed at the Multidisciplinary Meeting (MDM) of each center. The MDM evaluated each technique sample and how it contributed to the diagnosis. We categorized a biopsy as diagnostic only in those cases were the TBCB or TBLB findings were able to contributed to a definitive or high confidence diagnosis. Second, regarding the detection of pneumothorax, the study design limited this possibility. Fluoroscopy checks immediately after obtaining biopsies with each method allowed only immediate and not lagged cases of pneumothorax to be detected. Nonetheless, we only observed one case of pneumomediastinum, observed two hours after the procedure and associated with both techniques. Our results reflect a number of cases of pneumothorax after TBCB that is consistent with most published series [7]. This result may reflect the characteristics of the included patients, as only patients with heterogeneous and not only fibrotic interstitial patterns were included. As recommended by different authors, all biopsies were fluoroscopy-guided, with the conventional biopsy forceps and cryoprobe positioned 1–2 cm from the visceral pleura. Studies describing a higher pneumothorax rate include more patients with interstitial pneumopathies and fibrotic patterns and a greater number of subpleural biopsies (<1 cm) [17, 19]. Finally, the number of biopsies taken is a limitation, but published studies vary as to the number of samples harvested and the optimal number for diagnosing ILD has not been established [33]. Consistent with our interpretation of the literature, we stipulated that at least 3 transbronchial samples should be taken with each biopsy method and that more would be taken if patient safety and tolerance allowed. Furthermore, we used fluoroscopy to locate and sample in the region identified by HRCT to be most involved and samples came from different segmental bronchi.

In conclusion, our multicenter study findings confirm that for ILD diagnoses, compared to conventional TBLB the information provided by TBCB in a multidisciplinary setting increases the diagnostic yield for histologically complex entities. We suggest that TBCB is a safe and useful technique that merits inclusion in the diagnostic algorithm for ILD.

## Supporting information

S1 Checklist (DOC)

S1 File (XLSX)
